# THE IMPACT OF CULTURE ON CIVIC ENGAGEMENT OF AGING ASIAN IMMIGRANTS: FINDINGS FROM A MIXED STUDY IN EDMONTON, CANADA

**DOI:** 10.1093/geroni/igae098.0627

**Published:** 2024-12-31

**Authors:** Hongmei Tong

**Affiliations:** MacEwan University, Edmonton, Alberta, Canada

## Abstract

As a well-known immigrant-receiving country, Asian immigrants constitute most immigrants in Canada. However, the understanding of cultural diversity and intra-cultural similarities and differences among aging immigrants’ civic activities is limited. A mixed-methods study was conducted to examine civic participation experienced by Filipinos, Indians, and Chinese, since they are the three largest ethnocultural communities in Edmonton. Thematic analysis related to research questions was used for data analysis. Findings show diversity in the understanding of civic engagement and willingness, and their understanding and engagement in civic activities are affected by culture-related factors such as country of origin, time since immigration, citizenship status, greater official language proficiency, and pre-migration participation in activities. Public policies, such as immigration and integration policies (e.g., multiculturalism, diversity, and social inclusion), also influence immigrants’ civic participation. The findings suggest that cultural diversity should be considered in promoting civic activities among aging immigrants.

